# Joint Center Estimation Using Single-Frame Optimization: Part 2: Experimentation

**DOI:** 10.3390/s18082563

**Published:** 2018-08-05

**Authors:** Eric Frick, Salam Rahmatalla

**Affiliations:** 1 Center for Computer-Aided Design, College of Engineering, The University of Iowa, Iowa City, IA 52242, USA; eric-frick@uiowa.edu; 2 Department of Civil and Environmental Engineering and Center for Computer-Aided Design, College of Engineering, The University of Iowa, Iowa City, IA 52242, USA

**Keywords:** motion capture, inertial sensors, optical markers, joint center, soft tissue artifact

## Abstract

Human motion capture is driven by joint center location estimates, and error in their estimation can be compounded by subsequent kinematic calculations. Soft tissue artifact (STA), the motion of tissue relative to the underlying bones, is a primary cause of error in joint center calculations. A method for mitigating the effects of STA, single-frame optimization (SFO), was introduced and numerically verified in Part 1 of this work, and the purpose of this article (Part 2) is to experimentally compare the results of SFO with a marker-based solution. The experimentation herein employed a single-degree-of-freedom pendulum to simulate human joint motion, and the effects of STA were simulated by affixing the inertial measurement unit to the pendulum indirectly through raw, vacuum-sealed meat. The inertial sensor was outfitted with an optical marker adapter so that its location could be optically determined by a camera-based motion-capture system. During the motion, inertial effects and non-rigid attachment of the inertial sensor caused the simulated STA to manifest via unrestricted motion (six degrees of freedom) relative to the rigid pendulum. The redundant inertial and optical instrumentation allowed a time-varying joint center solution to be determined both by optical markers and by SFO, allowing for comparison. The experimental results suggest that SFO can achieve accuracy comparable to that of state-of-the-art joint center determination methods that use optical skin markers (root mean square error of 7.87–37.86 mm), and that the time variances of the SFO solutions are correlated (r =  0.58–0.99) with the true, time-varying joint center solutions. This suggests that SFO could potentially help to fill a gap in the existing literature by improving the characterization and mitigation of STA in human motion capture.

## 1. Introduction

Methods for human motion capture continue to improve in both accuracy and practicality, and as a result, motion capture is now being used in a multitude of areas, including healthcare, military, industry, sports, and animation [1,2,3,4,5]. Though many methods of motion capture have been developed (e.g., electromagnetic, mechanical/goniometric, radiographic), optical (marker-based) and inertial methods are currently among the most popular. Marker-based motion capture is the current gold standard of the industry because of its rich literature background, low level of invasiveness, and accuracy [6,7].

A driving parameter of human motion capture is joint center location, from which joint angles can be calculated. This has led to the development of many methods for its estimation. Said methods can be divided into two broad categories—predictive and functional. Predictive methods, such as the Harrington equations, apply regression analysis to subject-specific anthropometric data to estimate joint center location [8]. Conversely, functional methods use subject-specific motion data to determine joint center location. Marker-based methods are typically based on sphere-fitting or a transformation technique. Sphere-fitting methods use optimization to find the radius that best fits the measured marker trajectories [9]. Transformation-based methods assume the inter-marker distance to be fixed, project the data into a common coordinate system, and determine an optimal joint center from the projections [9]. Inertial methods, which are based on measurements of translational acceleration and rotational velocity, have also been developed for joint center determination. In these methods, optimization or regression is typically applied to the equation for translational acceleration on a rotating body, treating the position vector as the design variables [10,11,12]. A recent work by Olsson and Halvorsen investigated how the different methodologies by which this acceleration equation can be expressed and optimally solved impact the estimated location of the joint center [13]. The investigation was based on experimental data (no simulation of STA) and numerical simulation (where STA was applied via the model proposed by Camomilla et al. [14]). A thorough examination of the results was provided, offering significant insight into how each mathematical approach interpreted the inertial measurements. The overall findings suggested that formulating the cost function as the sum of the squared errors and as the sum of absolute errors were each viable approaches. The sum of squared errors converged faster than the sum of absolute errors, but was less robust to outliers, such as those resulting from simulated STA [13].

There are joints in the human body that can be reasonably approximated as hinges (single-degree-of-freedom), and several methods are available for calculating the axis about which the joint articulates. A joint axis is defined by a point and a unit direction vector passing through it. Marker-based methods for calculating the joint axis are similar to the sphere-fitting and transformation methods used for joint center identification, but are adapted to reflect the two fewer degrees of freedom (e.g., cylinder-fitting) [15]. Inertial methods for finding the joint axis by exploiting joint kinematics have also been developed [10,16], including a method for identifying the paired joint axes of a two-degree-of-freedom joint, such as the elbow [17]. 

One of the greatest obstacles in human motion capture is posed by soft tissue artifact (STA), which occurs when the skin, muscle, and fat tissue to which a marker or sensor is mounted moves relative to the underlying bone. This motion is often modeled in the literature as a superposition of relative translation, relative rotation, homothety, and shearing, and has been investigated by many methods, including principal component and modal analysis [18,19,20,21,22]. Typically, the rigid components (relative translation and relative rotation) are assumed to represent the majority of STA and are the components targeted for mitigation [21,23,24]. However, recent research using a higher marker density (40 markers compared with the typical 4–6) suggests that mitigation of STA requires more than compensation of the rigid components, and the non-rigid component of STA sometimes requires more than homothety and shearing to be fully described [25]. 

Until recent years, validating joint center estimations has been either impossible or prohibitively impractical. Fortunately, recent advances in radiographic imaging have facilitated its use in human motion capture. A recent article [18] compared range of motion and joint angles for the hip as determined by marker-based functional methods and dual fluoroscopy, the latter of which is a radiographic method validated to bias and precision less than 1 mm and 1° [26]. The hip joint range of motion calculated from dual fluoroscopy data was found to differ from that calculated from skin markers by up to 21.8° (internal/external rotation). Furthermore, relative to dual fluoroscopy, the skin marker calculations exhibited errors in internal/external rotation, abduction/adduction, and flexion/extension of up to 5.8°, 0.6°, and 1.9°, respectively [18]. These differences suggest that the literature would benefit from a new method able to improve the mitigation of STA.

A recent article proposed the use of single-frame optimization (SFO) [14], a method for calculating a time-varying vector relating an inertial measurement unit (IMU) to its corresponding joint center in the presence of STA. SFO is intended to build on the work of Olsson and Halvorsen [13], where the impact of STA on inertially determined joint center locations was investigated, by attempting to mitigate STA via frame-specific direct calculation rather than robustness of cost function. This is accomplished by processing the translational acceleration and rotational velocity measured by the IMU. The method was verified by several cases of planar numerical simulation, including one where the simulated STA exhibited multimodal behavior. The purpose of this article is to physically recreate this simulation and to experimentally compare the SFO solution with a marker-based solution. A custom apparatus was built to provide a planar pendulum with a stationary joint axis, and vacuum-sealed, raw animal tissue was used in the simulation of STA. The apparatus was instrumented with two IMUs, each with a corresponding set of optical markers. One IMU was mounted on the meat, and the other was rigidly attached directly to the pendulum. The recorded data was sufficient to calculate independent time-varying joint center solutions, one by SFO and one from the marker data, allowing SFO to be compared with the marker solution. 

## 2. Methods: Experimentation

### 2.1. Review of SFO Methodology

Single-frame optimization is an algorithm that allows the location and orientation of an inertial sensor relative to its corresponding joint center to be determined uniquely at each time frame in the inertial sensor’s local coordinate system. The calculated locations and orientations define vectors that optimally satisfy the SFO cost function (Equation (1)) for the inertial data corresponding to each frame.

(1) 0 = ‖a − (α × r + ω × (ω × r)) − g‖

Here, a denotes the measured translational acceleration; ω denotes the measured rotational velocity; α denotes the rotational acceleration (calculated by numerical differentiation of ω); g is the scalar value of gravitational acceleration; and r, the design variable, denotes the joint center location vector. The formulation of Equation (1) is valid and complete only if it can be assumed that the joint center is undergoing negligible acceleration. In terms of human motion capture, this assumption may often be unreasonable, which would negatively impact any results achieved via Equation (1). Further work is required to make the SFO process independent of this assumption and more practically applicable in human motion capture.

The SFO methodology is summarized briefly here, and the reader is referred to Part 1 of this work for more details [12]. The first step in the SFO process is measurement and recording. Inertial data (tri-axial translational acceleration and rotational velocity values) are gathered by an IMU attached to a link rotating about some fixed point. The link or sensor attachment may allow for motion of the inertial sensor relative to the fixed point (joint center), such as relative rotation or relative translation. The second step is to examine the dataset and find a joint center solution that best fits the measurements as a whole. This is accomplished via the variance minimization method, which applies optimization individually to each frame in the dataset. This process is nested within another optimization process, in which the initial conditions used in the frame-by-frame optimization process act as the design variables, and the variance of the resulting array of joint center vector estimates constitutes the cost function. In this manner, the set of joint center vector initial conditions that produce the greatest number of stable optimization solutions is identified. This best-fit initial condition vector is then used in SFO’s third step, the final frame-by-frame optimization process. The best-fit vector acts as initial conditions for the optimization of the first frame. The resulting solution then acts as the initial conditions for the next frame’s optimization. More specifically, the initial conditions for each frame are defined recursively as the solution from the previous frame. The fourth step in SFO is to assemble the time series of joint center solutions into an array defining time-varying vector. This array is a linearized representation of the joint center vector, including the effects of STA. A lowpass filter and moving average filter are then applied to the array, yielding the final time-varying solution.

**Figure 1:**
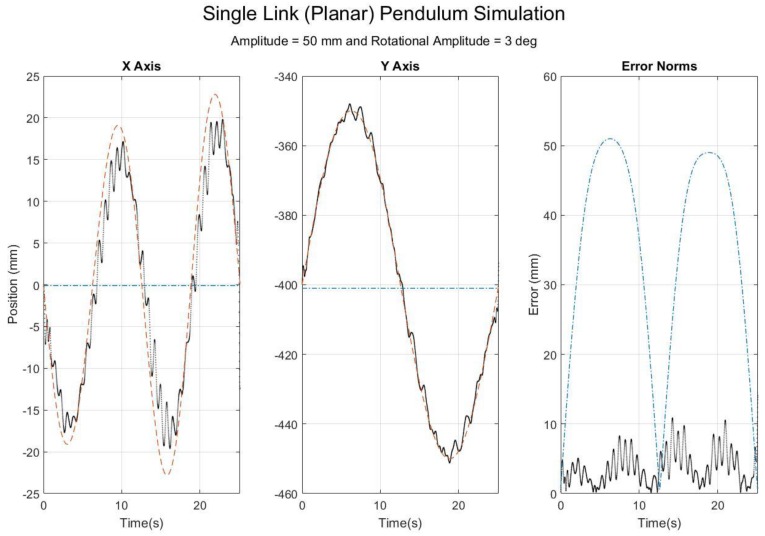
Results for planar, single-link simulation with translational and rotational soft tissue artifact (STA) added. The plotted data are expressed within the local frame of the inertial sensor. The red dashes refer to the theory solution, the black dots to the time-varying single-frame optimization (SFO) solution (as applied to the simulated inertial data), and the blue dash-dots to a static joint center solution [16].

To facilitate understanding of SFO’s operation and the meaning of a time-varying joint center realization, a new case is numerically simulated via the methodology explained in Part 1 (Frick, 2018), and the results are presented and discussed. The simulation generates rigid body motion, adds simulated STA, calculates corresponding inertial measurements, and then applies SFO to the simulated inertial data to see how effectively the non-rigid STA motion is captured (Figure 1).

The plots of Figure 1 are expressed within the local frame of the simulated inertial sensor, and thus any changes in the solution value from the rigid body parameters (0 mm in the *X* axis and −400 mm in the *Y* axis) are the direct result of the simulated STA. For reference, the simulated translational STA was 50 mm in amplitude, and the rotational STA was 3° in amplitude. The SFO results show that, just as the theoretical joint center solution changes over time, so does the SFO solution, exemplifying the concept of a time-varying joint center solution. In contrast, the other method [16] determines a single joint center vector that is optimal for the dataset as a whole. The time-varying SFO solution is able to track the motion because of STA, producing a solution highly correlated with the theoretical solution ($r_{X}=0.9757$ and $r_{Y}=0.9988$ for the results in Figure 1). This tracking produces a root mean square error (RMSE) lower that than of the static solution method (here, the RMSE is 4.4 mm for SFO and 38.4 mm for the other method, respectively).

### 2.2. Experimental Overview

**Figure 2:**
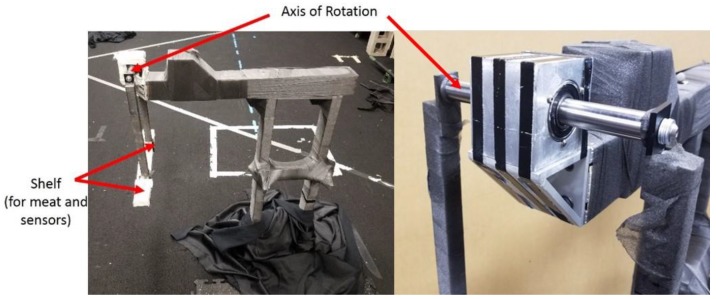
The experimental setup. The left image is of the overall setup, and the right is a close-up of the block-bearing apparatus (the tape used to mitigate reflection has been removed). The articulating rod defining the axis of rotation is labeled as such. The shelf is a flat part of the pendulum designed to facilitate attachment of the meat, inertial measurement unit (IMU), and optical markers.

The experiment described herein is designed to test the capability of SFO in predicting the joint center locations of a custom-built planar pendulum using an IMU non-rigidly attached to the pendulum (Figure 2). Inertial sensors and optical markers were used in the experiments to measure the angular velocity, translational acceleration, and position during experimentation. Single-frame optimization was applied separately to the inertial data from both rigidly and non-rigidly mounted sensors, and the resulting time-varying joint solutions were compared with a marker-based solution. Marker data was gathered via 15 Eagle-4 Motion Analysis cameras, calibrated pre-experiment such that the average spatial error was approximately 0.5 mm with a standard deviation of approximately 0.75 mm [27]. The SFO results from rigidly mounted IMU were primarily used for debugging purposes.

### 2.3. Pendulum Apparatus

A planar pendulum apparatus (Figure 2) was constructed, primarily with pieces from the 8020 Erector set, as seen in Figure 2 [28]. The rod serving as the axis of rotation interfaced with the rest of the apparatus via bearings, and the rod’s ends were tapped, allowing a pendulum with a length of approximately 425 mm and an incorporated shelf to be attached. All reflective surfaces that could reasonably be covered were covered in athletic pre-wrap or athletic tape to improve marker measurements.

**Figure 3:**
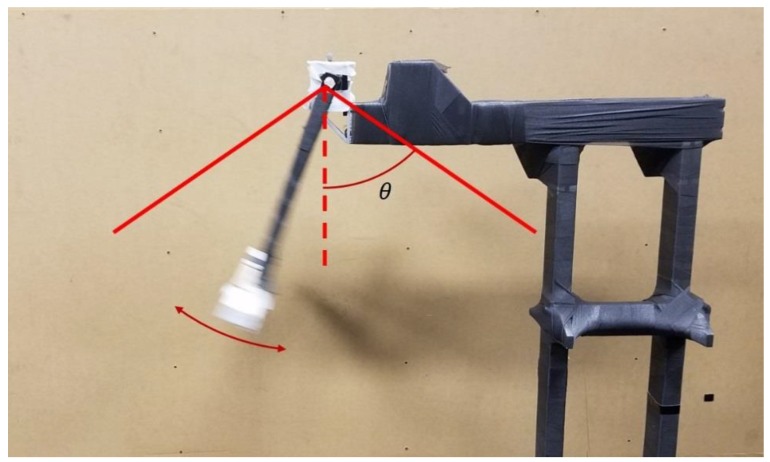
The range of motion achieved by the pendulum articulation. The release height (θ) was approximately 60° for all trials and would swing through nearly 120°.

During the experiment, the pendulum’s motion was generated by raising the pendulum shelf via articulating the pendulum such that part of the pendulum made contact with the pendulum’s stand, reaching an angle of approximately 60° with respect to the vertical. The pendulum was then released and allowed to swing freely, articulating through approximately 120° in the first swing, as can be seen in Figure 3, where θ denotes the release angle.

### 2.4. Simulation of STA

**Figure 4:**
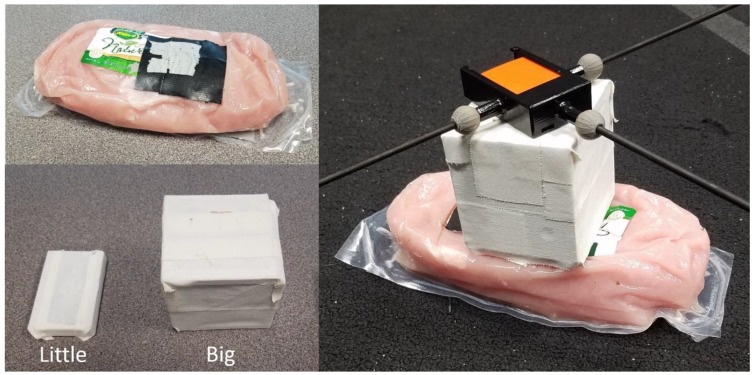
The vacuum-sealed turkey breast tenderloin (top left). The meat weighed ~680 g and had dimensions of approximately 215 by 115 by 45 mm. The blocks (referred to as “little” and “big”) used to improve the simulation of STA can be seen at lower left. The right-side image shows how the blocks were placed to amplify the relative motion of the sensor to the pendulum so as to improve the simulation of STA.

The non-rigid attachment of the IMU to the pendulum was a vital component in the experimental design. Given the propensity for ex vivo replications to poorly represent the STA of human motion [29], the goal for said attachment was to replicate STA as realistically as possible. The STA was simulated via a raw, vacuum-sealed turkey breast tenderloin (Figure 4, top left). What will be referred to as the bottom side of the meat (the side flush with the ground in Figure 4, top left) was rendered flat by the semi-rigid packaging used there. The packaging covering the rest of the meat was less thick and far more flexible. Double-sided duct tape was used to attach the bottom side of the meat to the top of the pendulum’s shelf. In the same manner, the inertial sensor could be attached to the top side of the meat. Note that a layer of Gorilla brand tape was applied to the top of the meat as an intermediate layer. 

The aforementioned steps achieved the goal of securely connecting the IMU to the rigid pendulum through the meat medium. Once attached, the magnitude of relative motion could be controlled by varying three parameters: pendulum release height, amount of weight attached to the inertial sensor, and the location of said weight’s center of mass. For simplicity, the pendulum’s release height was kept constant for all tests, and the parameters for the center and amount of mass were convolved via use of different solid blocks (Figure 4, bottom left), placing them between the meat and the sensor. This provided a method of simulating STA that involved actual biological tissue and could be tuned via a simple and quantifiable parameter (block choice). The mass and dimensions of each block are listed in Table 1. 

### 2.5. Pendulum Instrumentation

**Figure 5:**
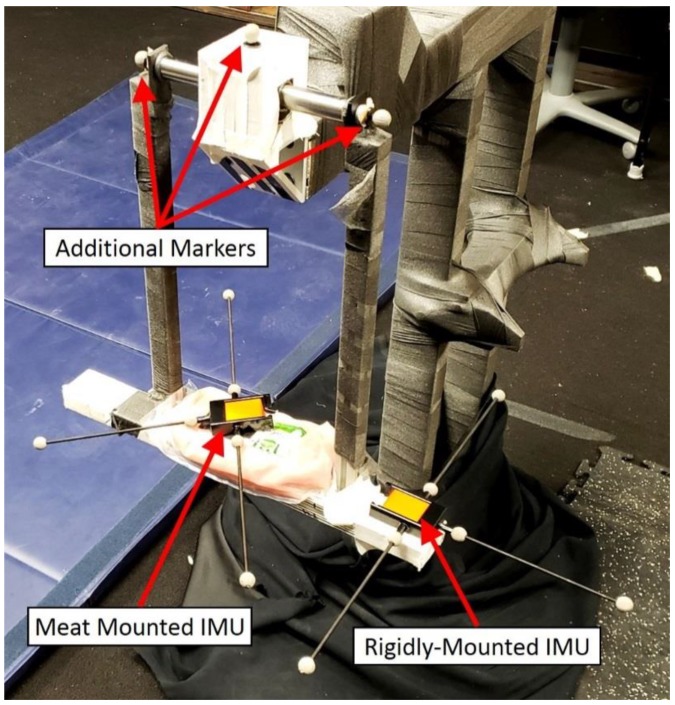
The pendulum as set up for experimentation. The IMUs and markers were placed such that the axis of rotation, IMU location, and IMU orientation could be calculated independently by both the optical and inertial systems.

**Figure 6:**
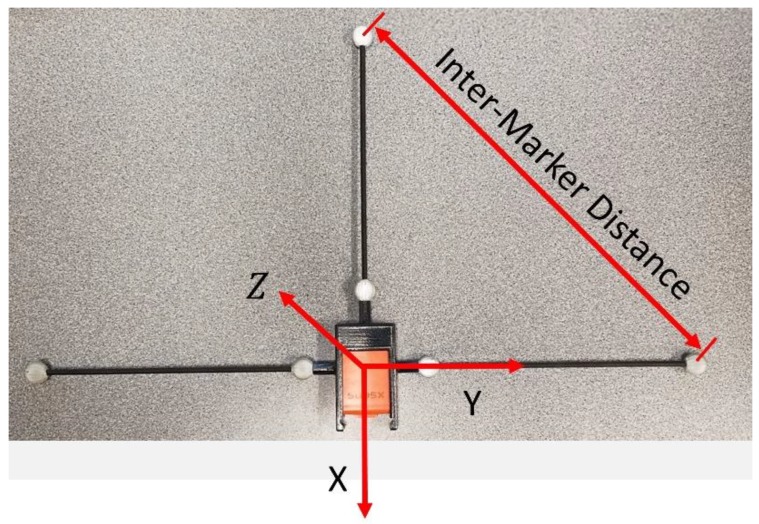
An inertial sensor inside a marker adapter. The coordinate system generated from the markers is denoted by the arrows and defines the IMU’s local coordinate system. Note that the offset vector determined by the accelerometer’s location within the IMU has not yet been applied to the origin of the coordinate system shown.

The pendulum apparatus was instrumented as seen in Figure 5. Two inertial sensors (XSENS MTws [30]) were placed on the pendulum—one rigidly mounted to the pendulum and one non-rigidly mounted to the pendulum (via packaged raw meat). Six markers were attached to each sensor (Figure 5) via a custom IMU-to-marker adapter (Figure 6). In addition, two markers were placed on the ends of the articulating rod (to provide an estimate for the axis of rotation) and one on top of the housing-block (to provide a reference location for the calculated joint center values). The sample rates for the inertial and marker data were 20 Hz and 100 Hz, respectively, requiring the marker data to be down-sampled to achieve a common time step. The inertial sample rate was lower because the sensor’s wireless communication outputs at the highest frequency it can provide over the entire recording. The measurement process of each system was initiated manually, resulting in a small but significant time offset between them that required correction. These processes of down-sampling and time synchronization are described in Section 3.2. 

This article utilizes three different coordinate systems, with the intent of allowing data and results to be presented in their most understandable form. The first utilized coordinate system is the global coordinate system (determined by the calibration of the optical marker system), which is denoted by X, Y, and Z. The second coordinate system is the local coordinate system of the meat-mounted IMU, which is denoted by $\bar{X}$, $\bar{Y}$, and $\bar{Z}$. The third coordinate system is the local coordinate system of the rigidly mounted IMU, which is denoted by $\hat{X}$, $\hat{Y}$, and $\hat{Z}$. The global coordinate system is used as the overall reference frame; the meat-mounted IMU’s coordinate system is used to express joint center vectors; and the rigidly mounted IMU’s coordinate system moves with the pendulum, making it ideal for expressing characteristics of the simulated STA.

### 2.6. Experimental Protocol

The experiments began by articulating the pendulum such that it made contact with the base of the apparatus, producing an initial angle relative to the vertical axis of approximately 60°, as discussed in Section 2.3. Recording by the optical and inertial systems was initiated, the pendulum was released, and recording continued until the pendulum had slowed appreciably (around 40 s). This was done for two cases, one where the little block was placed in between the meat and the IMU, and one where the big block was placed between them (Figure 4, right). One trial was performed for the small block, and a second trial was performed for the big block, each lasting approximately 39 s.

Single-frame optimization operates on the assumption that the STA-induced motion is negligible relative to the bone (or real) motion, and it can produce erratic results if this assumption is not adequately satisfied. At the beginning of each trial, the mechanical vibrations and non-planar motion that inevitably result from a manual release of the pendulum need to dissipate; otherwise, they will contaminate the inertial data (the acceleration measurements in particular). At the end of the trial, the pendulum’s velocity will have decreased, and the ratio of STA and sensor noise to real motion can become small enough that the optimization’s stability is impacted. To address this, the first five seconds of measurements were trimmed from the data, as were any measurements made after 35 s. This left 30 s of useable data.

## 3. Methods: Data Processing

### 3.1. Relating Optical and Inertial Frames

To compare the SFO results with optical markers, the marker-based calculation must be able to both locate the inertial sensor’s accelerometer (the location to which SFO makes its calculations relative) and determine the transformation matrix relating the IMU’s local coordinate system to the marker’s global coordinate system. To accomplish this, a custom adapter was created (Figure 6). A double cross-product approach was applied to the three most distal markers [31] to define a local coordinate system (and therefore the required transformation matrix). The local coordinate system was calculated such that it mirrored the IMU’s local coordinate system (Figure 6). After determining the rotation matrix, the three proximal markers were used to identify the location of the IMU’s accelerometer. The accelerometer is not centrally located within the IMU; therefore, the IMU’s top casing was removed and a micrometer was employed to measure the three vector components required to relate the crossing point of the three proximal markers to the accelerometer. 

The adapter’s distal markers were used to calculate the orientation of the IMU. The inter-marker distance impacts the accuracy of the angles calculated from the marker position (Figure 6), with the error magnitude determined by the accuracy of the optical system’s calibration error. After considering the accuracy of this calibration (~0.5 mm residual *w*/standard deviation ≈0.75 mm), the length of the inter-marker distance (of the distal markers) was chosen to be approximately 300 mm (the threaded rods in the adapter were cut to reflect this). Based on these values, the expected error in the calculated orientation would, when projected over the approximately 300–400 mm link length, be approximately ±2 mm. Note that the projection length is not 425 mm (the length of the pendulum) because the meat and blocks raise the IMU closer to the joint axis.

### 3.2. Data Synchronization 

The marker data was sampled at 100 Hz, and the inertial data was sampled at 20 Hz; therefore, resampling was required to allow for direct comparison. As down-sampling is more robust than up-sampling, the marker data was down-sampled to 20 Hz. The power spectral density was calculated for all utilized data sets, in terms of the accelerometer, gyroscope, and optical components. The results showed that, in all cases, 99.9% or more of the signal power was contained at or below 2 Hz. This ensures that less than 0.1% of the frequency content was located at or above 20 Hz, and any degradation of the data resulting from down-sampling can be assumed to be negligible. Resampling was accomplished via MATLAB’s “resample” function, which applies an antialiasing finite impulse response lowpass filter to the data to compensate for the filter-induced delay. Once the sample rates were matched, time synchronization was required. This was accomplished via the MATLAB function “alignsignals”, which leverages the cross-correlation to find the delay that optimally aligns the signals in time [32]. Such alignment requires that both signals are in comparable form; therefore, the position data gathered by the markers and the inertial data gathered by IMUs were not directly amenable to such alignment.

To achieve a comparable form between the marker and the inertial data, both were converted to Euler angles before alignment. The marker-based Euler angles were calculated directly from the marker-determined rotation matrices, and the inertial data was converted to Euler angles via a recursive gradient descent optimization process that uses gravity measurements to mitigate drift as a result of integration of gyroscope bias [33]. The Euler angles (marker and inertial) were determined according to the common *ZYX* rotation sequence. With both data streams converted to Euler angles, the optimal time offset could be calculated and then applied directly to the raw marker and inertial data. Therefore, by the two processes expounded upon in this section, the inertial and marker data were made suitable for direct comparison.

### 3.3. Marker-Based Joint Axis

Pendulum articulation was planar, so a unique joint center could not be determined, only a unique joint axis. To this end, optical data from the 15 markers were gathered, and the global coordinate system was set up (in calibration) such that its *X* axis was roughly parallel (through manual placement) to the joint axis. As seen in Figure 5, six infrared-reflective markers were interfaced with each inertial sensor. The position data from the six markers corresponding to the rigidly attached sensor were used to determine the pendulum’s axis of rotation. This was accomplished by a cylindrical arc optimization approach similar to that proposed by Gamage and Lasenby [34] and was implemented using the Matlab Optimization Toolbox [35]. The resulting solution consisted of a direction vector and a point, which together fully defined the pendulum’s joint axis.

### 3.4. Marker-Based Joint Center

This experimentation was performed on a planar pendulum (single-degree-of-freedom); therefore, any point on the pendulum’s axis of rotation is a valid joint center solution. To remove this ambiguity, the joint center was defined as the instantaneous point on the joint axis of the pendulum that is shortest in distance from the origin of the sensor’s local coordinate system (defined by its corresponding markers), meaning that it was uniquely calculated and defined at each time step. The process of calculating these instantaneous joint centers required a projection process [10], defined as follows:

(2) $$JC_{Inst}=JC_{o}-JA\left(JC_{o}\cdot JA\right)$$

The term $JC_{Inst}$ denotes the instantaneous joint center calculated as the point on the joint axis closest to the sensor, $JC_{o}$ refers to the point on the joint axis as calculated by joint axis optimization, and JA denotes the previously calculated joint axis (post normalization). Finally, “·” denotes the scalar, or dot product. Note that all of these terms are expressed in the local coordinate system of the IMU/marker adapter to which they correspond.

**Figure 7:**
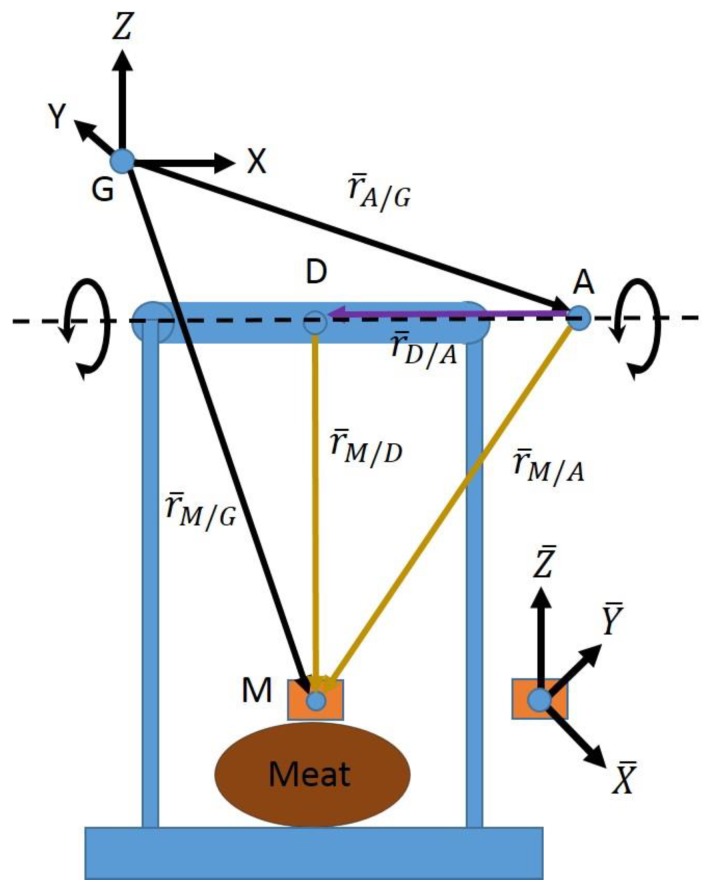
The pendulum apparatus with the meat (oval) and the corresponding sensor (block on top of oval). The three vectors at point G represent the axes of the global coordinate system. The bars on top of the vectors denote that they are expressed in the local coordinate system defined by the markers attached to the meat-mounted IMU (defined per Figure 6). The axes of the local coordinate system are shown at right, and the origin of the local coordinate system coincides with point M. Point A represents the point along the joint axis determined by the optimization process for the corresponding frame. Finally, point D represents the to-be-calculated point along the joint axis to which point A will be projected. The resulting location of point D acts as the instantaneous joint center for the corresponding data frame.

Figure 7 graphically explains the kinematic implementation of Equation (2). Point G is the origin of the global marker-based coordinate system, within which the locations of point A (the point on the joint axis identified by the marker optimization or SFO) and point M (location of the meat-mounted IMU’s accelerometer) are known. The vectors defining these points are projected into the local coordinate system of the IMU via a transformation matrix, yielding ${\bar{r}}_{A/G}$ and ${\bar{r}}_{M/G}$ from which ${\bar{r}}_{M/A}$ ($JC_{o}$ in Equation (2)) can be calculated via subtraction. Furthermore, the direction of vector ${\bar{r}}_{D/A}$ (JA in Equation (2)) is the same as the joint axis, which is also known in the global system and can be projected into the local coordinate system. If one were to express Equation (2) in reference to Figure 7, Equation (2) could be expressed as follows:

(3) $${\bar{r}}_{M/D}={\bar{r}}_{M/A}-\frac{{\bar{r}}_{D/A}}{\left\|{\bar{r}}_{D/A}\right\|}\left({\bar{r}}_{M/A}\cdot \frac{{\bar{r}}_{D/A}}{\left\|{\bar{r}}_{D/A}\right\|}\right)$$

Equation (2) provides an analytical method for determining the shortest vector ($JC_{Inst}$). The values of $JC_{o}$ are determined separately by the marker data and SFO. Equation (2) is applied to both, allowing for an objective comparison.

### 3.5. STA Quantification

The relative translation due to STA was calculated by subtracting the location of the rigidly mounted IMU accelerometer from that of the meat-mounted IMU accelerometer (in the global XYZ coordinate system), and projecting the resulting vector into the pendulum-mounted coordinate system ($\hat{X}\hat{Y}\hat{Z}$). Finally, to more clearly show the relative motion, the relative vector was shifted in all three components such that their values at the first time step were zero. 

To calculate the rotation of the meat-mounted sensor relative to the rigidly mounted sensor, the rotation matrices defining the meat-mounted IMU in the global coordinate system (XYZ) were projected into the coordinate system of the rigidly mounted IMU ($\hat{X}\hat{Y}\hat{Z}$). The orientation of the rigidly mounted IMU’s coordinate system was defined by the pendulum’s orientation, meaning that any rotation still present after the projection was a result of the meat-mounted IMU moving relative to the pendulum. The projected rotation matrices were then converted to Euler angles (*XYZ* sequence).

### 3.6. Experiment-Specific Methodology

Processing the experimental data gathered in this work with SFO required addressing two issues not directly addressed in the simulation work [12]. First, the parameters for data filtering require specification. A fourth-order, bidirectional (effective order of 8) lowpass Butterworth filter with a cut frequency of 8 Hz was applied to the raw inertial data. A cut frequency of 8 Hz was chosen because the majority of the frequency content in human motion is often assumed to be contained below 6 Hz [36], and setting the 3 dB point at 8 Hz restricted the attenuation of data at 6 Hz and below to less than 0.01 dB. A moving average filter with a span of 0.5 s was also applied to the raw SFO results. Second, the time span over which the variance minimization method was applied must be specified. In the simulation [12], the entire dataset was considered. Here, however, only the time span from 0–5 s is used, reducing the computational requirements.

## 4. Results

**Figure 8:**
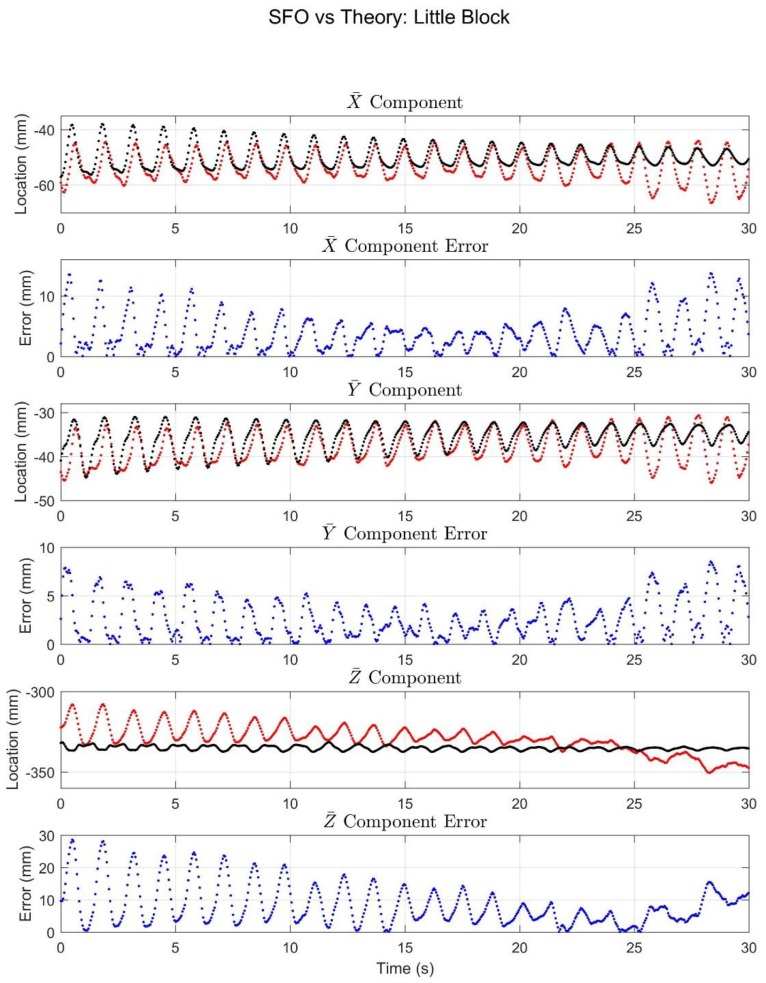
The results for the little block trial. The black and red (or light and dark) plots denote the marker and SFO joint center component solutions, respectively, in terms of $\bar{X}$, $\bar{Y}$, and $\bar{Z}$ components. The error plots show the difference between the two solutions.

The experimental results are displayed via graphical comparisons expressed in the $\bar{X}\bar{Y}\bar{Z}$ coordinate system, and the RMSE is tabulated for each case, as are the Pearson Correlation values relating the SFO solution to the marker solution for each component. The SFO results for the little block can be seen in Figure 8.

**Figure 9:**
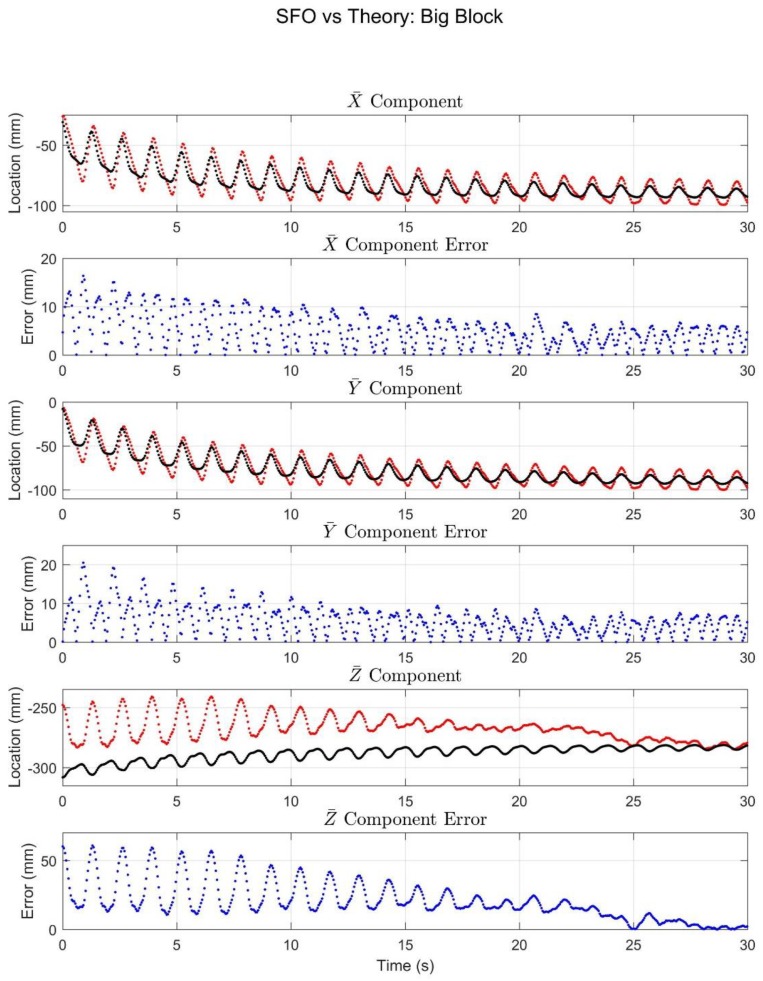
The results for the big block trial. The black and red (or light and dark) plots denote the marker and SFO joint center component solutions, respectively, in terms of $\bar{X}$, $\bar{Y}$, and $\bar{Z}$ components. The error plots show the difference between the two solutions.

The SFO results for the big block trial (Figure 9) behaved largely in the same manner as those of the little block. The major difference was that the components exhibited both a periodic motion and a slower drifting motion. The local $\bar{X}$ and $\bar{Y}$ components tracked both motion types well, but the accuracy of the $\bar{Z}$ component did decrease compared with the little block trial.

To facilitate understanding of the presented results, they have been quantified in Table 2. Specifically, the RMSE and correlation values have been expressed for each component, as well as the overall RMSE. The results for each block trial are subdivided into four time spans: 0–30 s (whole trial), 0–5 s (beginning), 10–15 s (middle), and 25–30 s (end). These subdivided results are shown because the conditions at each of the time segments differ, primarily in terms of magnitude of pendulum motion (it starts at its largest value and continually decreases with time). Though the trials were divided into four time spans each, there is no objective reason that greater or fewer subdivisions could not have been used. Four spans were chosen because, in the opinion of the authors, the subsequent analysis was detailed enough to offer insight into how the different types of motion could impact the SFO results, but not so detailed as to overwhelm the reader.

**Figure 10:**
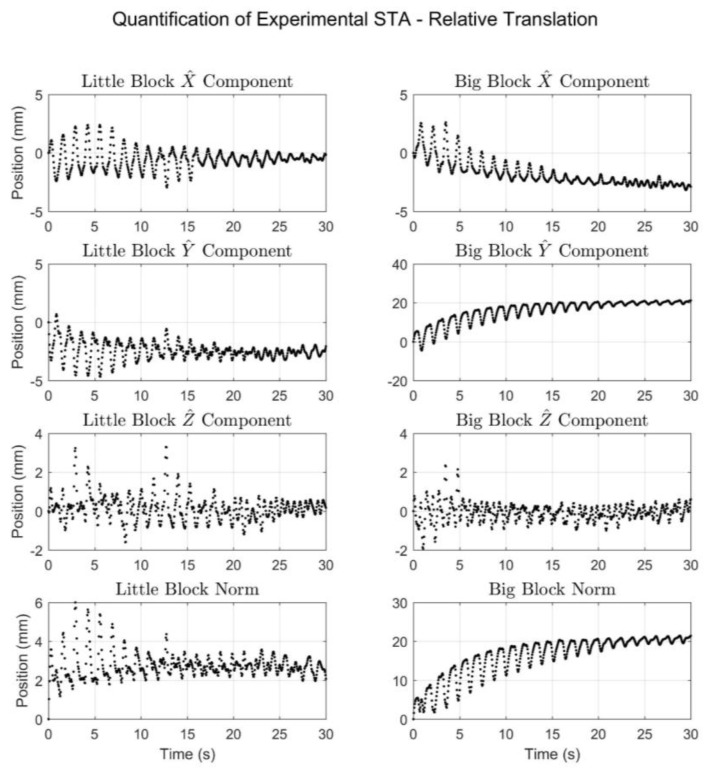
The translational motion of the meat sensor relative to the rigid sensor in terms of the local $\hat{X}$, $\hat{Y}$, and $\hat{Z}$ components and the norm for both the little block trial (**left**) and the big block trial (**right**).

**Figure 11:**
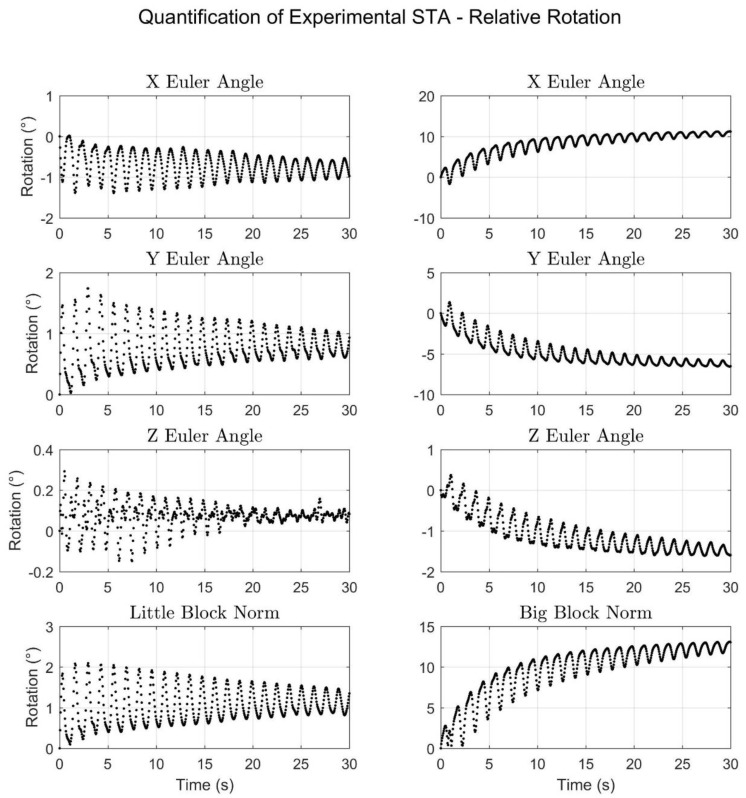
The rotational motion of the meat sensor relative to the rigid sensor shown as Euler angles (*XYZ*) and their corresponding norm for both the little block trial (**left**) and the big block trial (**right**). The results are expressed in the $\hat{X}\hat{Y}\hat{Z}$ coordinate system.

In terms of quantifying the experimentally produced STA, Figure 10 and Figure 11 display the motion due to STA in terms of translation and rotation, respectively, of the meat-mounted IMU relative to the rigid pendulum. Together, the figures show that despite single-degree-of-freedom input motion, the resulting simulated STA exhibited six degrees of freedom and also exhibited oscillatory behavior over short time scales, as well as a drift component (in the big block trial) over longer time scales. To facilitate understanding of the results shown in Figure 10 and Figure 11, range-of-motion metrics were calculated and are listed in Table 3. The overall range-of-motion values are the difference between the maximum and minimum values for the entire time period. The oscillatory range-of-motion values were defined as the maximum range of motion that occurred over a 1.5 s period. An interval of 1.5 s was chosen because each oscillation lasted approximately 1.25 s.

## 5. Discussion

The purpose of this article was to experimentally compare the SFO method with marker-based solutions under situations of simulated STA. The results of Figure 8 and Figure 9 suggest that under the simulated STA conditions, SFO exhibits error (relative to the marker-based solution) comparable in magnitude to state-of-the-art joint center determination methods [9,29]. Furthermore, a time-varying joint center realization was achieved (instead of a static realization) and was generally well-correlated with the marker-based solution. This suggests that SFO is capable of directly estimating the effects of STA in the time domain, and that it could potentially fill an important gap in the literature in terms of STA characterization and mitigation [25].

### 5.1. Interpretations of Results

According to a recent review, the most accurate method for hip joint center location determination was the geometric sphere fit method, which exhibited an average error of 11–21 mm (the 95% confidence interval for the greatest resulting error was 17.8–24.4 mm) when compared with radiographic methods [29,37,38,39,40,41], though such errors can be far greater and are impacted by things like activity type [29,42]. The errors of the presented motion trials are often larger than this, but SFO yields a time-varying, rather than static, joint center, which makes direct comparison somewhat speculative. The SFO results for the little block trial (Figure 8) exhibited small errors relative to the marker-based solution and were well-correlated with the marker-based solution in the local $\bar{X}$ and $\bar{Y}$ components. The relative motion in the $\bar{Z}$ component was small and was thus not expected to produce meaningful correlations. When interpreting the data shown in Figure 8 and Figure 9, it should be noted that the plotted data is expressed in the local coordinate system of the IMU, and the plots reflect only the relative motion of the sensor to the pendulum (meaning STA), not the overall motion. Therefore, the plots can be interpreted as showing how accurately SFO predicted the motion resulting from STA. Furthermore, in the authors’ opinion, the correlation values achieved by SFO offer strong evidence of SFO’s efficacy. Single-frame optimization is the only method known to the authors that has been used to calculate a time-varying joint center realization, and would thus be the only one capable of calculating a correlation relative to the true solution. Optical markers were used to calculate such a solution in this experiment, but this was only possible because the joint axis was fixed, which is unlikely to be a reasonable assumption in human motion.

The single-frame approach of SFO makes solution stability an issue of import, and the correlation values offer a quantifiable manner of showing SFO’s ability to capture the effects of STA. As can be seen in Figure 8 and Figure 9, the STA motion was most pronounced, especially with respect to the component magnitude, in the local $\bar{Y}$ axis, and the correlation values for said axis were high ($r_{\bar{Y}}\geq 0.75$, up to 0.99). These values are evidence that SFO is tracking the effects of STA. Examining the results of Table 2, specifically those of the subset time periods, it can be seen that, though they remain within what the authors consider to be an acceptable range, the RMSE and correlation values fluctuate based on which section of the data is considered. Each time period exhibited different motion characteristics, specifically, the magnitudes of pendulum motion, STA motion, and the ratio of the two motions varied between subsets. The differing results suggest that these parameters impact the efficacy of SFO, but the analysis required to characterize said efficacy is outside the scope of this article. Overall, the presented results suggest that SFO is capable of producing time-varying joint center solutions with enough accuracy to be potentially useful in motion capture analysis, and the time-variance of said solution is directly related to the STA contaminating the overall motion, as shown by the correlation values.

Finally, note that the simulated STA in the big block trial was, in general, several times larger than that of the little block trial, and this is reflected in the errors in Table 2, increasing from approximately 12 mm to 26 mm (total RMSE for the whole time span). This suggests that SFO can mitigate the effects of STA, but that its performance may decline as the amount of STA increases. As an example, the $\bar{Z}$ component and related error in the big block trial was large (max. error was 60.65 mm), however, the error decreased over the course of the trial (the mean $\bar{Z}$ error over the last 10 s was 8.91 mm), a trend mirrored by the $\bar{X}$ and $\bar{Y}$ components, though to a lesser extent. In terms of the component values’ fluctuations, the STA decreased over the course of the trial, which can be directly attributed to the decreasing amplitude of the pendulum’s swing over time (which is representative of damped sinusoidal motion). Furthermore, in the approximate time period of 10–20 s, the oscillation magnitude in the $\bar{Z}$ error rapidly diminishes. This suggests that there may be something akin to a threshold governing SFO solution accuracy and stability, that is, there is a certain magnitude of STA above which SFO’s stability and accuracy diminish and below which SFO’s stability and accuracy are relatively constant. This threshold-type behavior could also explain the apparent negative correlation between the SFO results and the optical solution for the first 15–20 s of the little and big blocks’ $\bar{Z}$ component plots, as the apparent negative correlation diminishes with time. Further work will be necessary to investigate the existence and nature of this threshold.

Based on findings from the literature, attempts to approximate STA with mechanical apparatus are typically unsuccessful, tending to produce errors an order of magnitude lower than errors for the same method when applied to a human [29,43]. The STA in the experiment performed for this work was simulated by using vacuum-sealed raw animal muscle tissue as the interface between the IMU and the rigid pendulum. It was reasoned that using real muscle tissue would ensure that the simulated STA was driven by the properties of a biological material. Originally, the intent was to remove the vacuum sealing around the meat before attaching the sensor. However, inspection revealed that the plastic wrapping acted similarly to human skin, smoothly sliding over the underlying meat. There was a small amount of fluid inside the packaging that lubricated the relative motion of the plastic wrap. Retaining the plastic also solved the problem of creating an attachment between the IMU, meat, and pendulum, as it was far more amenable to adhesives than raw meat. Overall, it was intended for the turkey tenderloin to approximate the muscle and fat components of STA (such as damping) and for the plastic sealing to approximate the skin components of STA (by encouraging shear motion). 

In preliminary motion trials, where the IMU was directly mounted to the meat, the configuration produced motion that visually appeared to reasonably simulate STA. However, in terms of the measured inertial values, there was little difference between the rigidly mounted IMU and the meat-mounted IMU, and the relative motion calculated from the optical markers was smaller than desired. The sinusoidal pendulum motion of the apparatus forces the majority of the observed STA to be the result of inertial effects, so it was reasoned that increasing the inertia of the meat-mounted IMU would increase the STA. Therefore, the little and big blocks were implemented to add mass to the sensors and amplify the inertial effects. Furthermore, the blocks increased the distance between the sensor and the point of articulation relative to the meat (the block–meat interface). This further amplified the motion of the sensors relative to the rigid pendulum. In sum, mounting the sensors directly to the meat did not adequately simulate the inertial effects of STA, so the inertial effects were amplified to more reasonable levels by introducing the little and big blocks.

A pivotal assumption of the SFO method is that the motion due to STA is small enough relative to the real motion that the optimization process can remain stable. Therefore, quantifying the STA present in the presented trials would be beneficial in quantifying the stringency of this assumption. As such, the STA in these trials was quantified in terms of both relative translation and relative rotation, which were expressed in the local coordinate system defined by the IMU rigidly mounted to the pendulum shelf (the $\hat{X}$, $\hat{Y}$, and $\hat{Z}$ coordinate system). The local $\hat{X}$ axis of this coordinate system was set to be parallel to the pendulum’s joint axis (within the limits of manual placement). 

The results exhibited by Figure 10 and Figure 11, and Table 3 provide an objective quantification of the simulated STA, which allows it to be contextualized via the available literature. A recent paper by Fiorentino et al. examined motion about the hip via skin markers and dual fluoroscopy [18]. Part of the analysis consisted of tracking the motion of the skin markers relative to anatomical landmarks identified radiographically on the pelvis and femur, allowing for direct calculation of the impact STA has on the position of skin markers. The mean relative change in marker position (in terms of linear distance) due to STA was calculated for six sites over four activities. For the level walking activity, the six site values ranged from 13–38 mm (at the posterior superior iliac spine and greater trochanter locations, respectively). Differences in experimental methodology make it untenable to directly compare the STA simulated herein with that measured by Fiorentino et al. [18]. However, the similarities between the two suggest that the simulated STA could be considered a reasonable approximation of true experimental STA.

To contextualize the relative rotation component of the simulated STA, a study that uses marker clusters (rather than lone markers) and compares the measurements with a radiographic method is required. The work of Grimpampi et al. satisfies these criteria and reported median values for orientation changes due to STA as 6.9° to 13.6° for its three cadaver subjects (clusters were formed from markers on the pelvis and thigh) [44]. A similar study by Barre et al. on 19 subjects with prosthetic knees (only the data from 10 were used for cluster evaluation) reported the average cluster orientation RMSE as 1.2° to 8.1° for the thigh [19]. As with the study by Fiorentino et al., direct comparison with these results is not permissible, but the values for this experiment (Table 3, right) are similar enough to suggest that they are reasonable.

The results from the big block trial as presented (Figure 9, Figure 10, right, and Figure 1, right) exhibit a drift component in addition to the oscillatory component. This drift was intended to simulate the effects of the potential “settling” of IMUs in inertial systems, where the IMUs embedded in the Lycra suit or mounted to the subject via straps tend to slowly shift during motion trials, potentially invalidating the pre-trial calibration. The drift could also be seen as a simulation of skin sliding during joint articulation. This could become relevant if the calibration was done at a certain joint angle (such as an anatomical position) and the motion predominately exhibited a different joint angle.

Comparing the results of Table 2 and Table 3, it can be seen that the error in the joint center estimation components is often greater than the translational STA. This would suggest the method is not effective if the simulated STA is restricted to relative translation. When making this comparison, however, the effect of relative rotation must also be considered. Relative rotation introduces localization error that is necessarily projected over the length of the joint center vector, amplifying its impact. Therefore, direct comparison of translational STA and joint center estimates is untenable, as the rotational STA must be accounted for. A potential method of overcoming this lack of direct comparability would be using the SFO results to isolate and remove the impact of STA. This would allow an estimate of the STA-free motion to be obtained, which could then be compared with a direct measurement of said motion. The complexity of such a calculation places it outside the scope of this article, though it may be explored in future work.

### 5.2. Limitations

The limitations of this experimental comparison mostly center on how closely the experiment represents human motion. The primary limitation is that the pendulum’s joint axis is stationary, which is unrealistic for human motion. Therefore, the results herein provide evidence for the validity of the SFO method, but this validity cannot yet be extended to the application of SFO to human motion. However, it should be noted that, at least to the knowledge of the authors, there is no method by which, in the presence of STA, a time-varying joint center vector solution can be determined by skin-marker methods, except in the case of a fixed joint center. In this regard, a fixed joint center was required to generate a time-varying joint center solution from optical markers, which is necessary to allow comparison with the SFO results. Future work will focus on adapting SFO to scenarios of non-stationary joint axes/centers, and thus will be more practically applicable to human data. Another limitation is the method by which STA was simulated, as such simulations often poorly reflect true STA [29]. The authors believe it is reasonable to assume that the vacuum-sealed meat exhibits material properties similar to those of the human tissue responsible for STA, but this does not account for the active nature of live muscle tissue during movement, such as contraction-induced conformation changes. Furthermore, the inertial effects of the simulated STA are dependent on the frequency content and amplitude of the pendulum motion, which is itself a simulation of human motion. However, these limitations center primarily on the quality with which each aspect of STA was simulated rather than on their absence, and, in the opinion of the authors, the experimental STA reasonably reflects the relevant characteristics of true STA. Furthermore, the authors believe that SFO’s linearization by the optimization approach provides a useful platform upon which SFO could potentially be developed into a method that is well-equipped to deal with the greater complexity of STA that would be encountered in human motion.

Another limitation is that the pendulum was articulated with one degree of freedom. Few human joints can be reasonably modeled in this manner (e.g., the knee), and many are often modeled as exhibiting two or three degrees of freedom (e.g., the elbow and shoulder, respectively). However, the authors do not expect that the accuracy of SFO will necessarily decrease when applied to joints with two or three degrees of freedom. It is expected that the additional axes of articulation would yield data that further constrained SFO’s cost function, potentially improving solution stability rather than degrading it. Furthermore, the pendulum motion was constrained to a single degree of freedom, but the simulated STA was unconstrained and exhibited six degrees of freedom. The application of SFO to two- and three-degree-of-freedom joints will be explored in future work.

Another potential limitation was the low sample rate of the inertial sensors (20 Hz), especially because, per the sensor specifications, two MTws can sample at rates of up to 100 Hz [45]. The primary reason for this lower sample rate was communication problems that emerged as a result of the experimental setup. The MTws are wireless sensors (with no option for onboard data storage, e.g., via Secure Digital memory card), and when communication with the receiver is interrupted, the transmitted data is automatically down-sampled to a rate at which a continuous data rate can be maintained. The locations of the pendulum apparatus, optical system, and data acquisition hardware interfered with the MTw communication, and 20 Hz was the fastest sample rate that could be reasonably achieved. At least 99.9% of the recorded motion’s frequency content (optical and inertial) occurred at or below 2 Hz, so sampling at 20 Hz should be fast enough to prevent loss of data. Further supporting this, the MTws sample at much higher rates internally (1800 Hz) to guarantee the capture of dynamic motion, and the data is filtered and down-sampled to the output rate [30].

As with all experimentation, a final limitation is the accuracy of the utilized hardware and experimental setup. For example, the calculations herein assume that the MTw casing is perfectly aligned with the internal sensor coordinate system. It is not expected that the experimental results were altered (for better or for worse) by imperfections in the experimental setup; rather, the sensitivity of RMSE values to such imperfections suggests that the correlation values may be a more reliable indicator of SFO’s efficacy than the RMSE values.

### 5.3. Comparison with Simulation

The article in which SFO was first introduced verified the method by numerical simulation [12]. The simulation results showed that SFO exhibited approximately one-half or less the error of the comparator method, and the SFO solutions were often highly correlated with the true motion (r>0.82) [12]. Direct comparison of the experimental results with the numerical simulation results could potentially be unreasonable, as the STA mathematically generated for the numerical simulation’s STA is likely different from that of the experiment. For example, the experimental STA exhibited six degrees of freedom instead of two, and the modes of the numerically simulated STA were based on literature-derived values, rather than determined by the coupling with the rigid body motion, as was the case in the experimental STA. However, despite the potential disparity between the numerically simulated and experimental STA, the experimental results exhibited characteristics similar to those of the simulation (e.g., correlation values, optimization stability). This suggests that the numerically simulated STA was at least somewhat representative of the experimental STA, and that the results of the numerical simulation likely have some predictive power as to how SFO will perform in an experimental situation. The experimental error (~8–38 mm) was greater than the simulation error (~0–8 mm), but the authors believe this to be reasonable given the ideal nature of the simulation. Furthermore, the experimental local $\bar{Y}$-axis correlation values ($r_{\bar{Y}}=$ 0.75–0.99) were very similar to the relevant corresponding values of the simulation (0.82–1.00). In the opinion of the authors, the experimental results fall well within the range expected from the simulation’s results.

## 6. Conclusions

A recently proposed method for mitigating the impact of soft tissue artifact on joint center location accuracy, single-frame optimization, was experimentally tested. Motion was generated by a single, rigid pendulum articulating about a fixed point, and soft tissue artifact was simulated by vacuum-sealed raw meat. The results, specifically the obtained correlation values, suggest that the single-frame optimization method is capable of using an optimization-based linearization approach to track the motion due to soft tissue artifact. The experimentally produced soft tissue artifact was quantified and discussed, as were the current limitations of the method.

## Figures and Tables

**Table 1:** Parameters (mass and dimensions) of the blocks used to improve the simulation of soft tissue artifact (STA).

Block	Mass (g)	Height (mm)	Width (mm)	Depth (mm)
Little	136	13	50	75
Big	496	80	80	70

**Table 2:** Numerical quantification of both motion trials (and time-based subsets). The root mean square error (RMSE) of the single-frame optimization (SFO) results relative to the marker-based solution is shown, as well as the Pearson correlation between the two solutions.

		Component RMSE (mm)					
Block	Time Period (s)	$\bar{X}$	$\bar{Y}$	$\bar{Z}$	RMSE (mm)	$r_{\bar{X}}$	$r_{\bar{Y}}$
**Little**	0–30	4.95	3.26	10.58	12.13	0.73	0.78
	0–5	6.02	3.93	15.38	16.98	0.69	0.75
	10–15	3.58	2.40	9.56	10.49	0.89	0.89
	25–30	7.07	4.66	8.50	12.00	0.98	0.96
**Big**	0–30	6.19	6.64	25.00	26.59	0.91	0.93
	0–5	9.23	10.12	35.29	37.86	0.85	0.87
	10–15	5.94	6.11	27.28	28.58	0.86	0.86
	25–30	4.17	4.69	4.74	7.87	0.99	0.99

**Table 3:** Values describing both the overall and oscillatory range of motion (ROM) due to the simulated STA in terms of relative translation and relative rotation for both the little and big block trials.

	Relative Translation (mm)		Relative Rotation (°)	
	Little Block	Big Block	Little Block	Big Block
Overall ROM				
*X* Component	5.3	5.8	1.4	12.9
*Y* Component	5.3	25.7	1.7	7.9
*Z* Component	4.9	4.3	0.4	2.0
Norm Component	6.0	21.4	2.1	13.1
Oscillatory ROM				
*X* Component	4.5	4.4	1.4	6.0
*Y* Component	4.8	13.3	1.6	4.3
*Z* Component	4.2	3.9	0.4	0.9
Norm Component	4.4	10.8	2.0	6.6
